# Hospital utilization among urban poor in Indonesia in 2018: is government-run insurance effective?

**DOI:** 10.1186/s12889-023-15017-y

**Published:** 2023-01-12

**Authors:** Ratna Dwi Wulandari, Agung Dwi Laksono, Rofingatul Mubasyiroh, Rika Rachmalina, Mara Ipa, Nikmatur Rohmah

**Affiliations:** 1 Faculty of Public Health, Universitas Airlangga, Surabaya, Indonesia; 2 National Research and Innovation Agency, the Republic of Indonesia, Jakarta, Indonesia; 3 Faculty of Health Science, Muhammadiyah University of Jember, East Java, Indonesia

**Keywords:** Government-run insurance, Health insurance, Hospital utilization, Healthcare evaluation, Health policy, Public health

## Abstract

**Background:**

An urban poor is a vulnerable group that needs government financing support to access health services. Once they are sick, they will fall deeper into poverty. The study aims to analyze the effectiveness of government-run insurance in hospital utilization in urban poor in Indonesia.

**Methods:**

The research analyzed the 2018 Indonesian Basic Health Survey data. This cross-sectional survey collected 75,970 participants through stratification and multistage random sampling. Meanwhile, the study employed hospital utilization as an outcome variable and health insurance ownership as an exposure variable. Moreover, the study looked at age, gender, marital status, education, and occupation as control factors. The research employed a binary logistic regression to evaluate the data in the final step.

**Results:**

The results show that someone with government-run insurance is 4.261 times more likely than the uninsured to utilize the hospital (95% CI 4.238–4.285). Someone with private-run insurance is 4.866 times more likely than the uninsured to use the hospital (95% CI 4.802–4.931). Moreover, someone with government-run and private-run insurance has 11.974 times more likely than the uninsured to utilize the hospital (95% CI 11.752–12.200).

**Conclusion:**

The study concluded that government-run insurance is more effective than the uninsured in improving hospital utilization among the urban poor in Indonesia. Meanwhile, private-run is more effective than government-run and uninsured in improving hospital utilization among the urban poor in Indonesia. Moreover, the most effective is to combine the kind of health insurance ownership (government-run and private-run).

## Introduction

An urban area is an area that has main non-agricultural activities with the structure of the function of the site as a place for urban settlements, concentration and distribution of government services, social services, and economic activities [1]. Vulnerability in urban communities is often caused by having a high population density and diverse livelihoods [2]. The main problem for urban residents is slum settlements resulting from poverty. Poverty is deprivation, namely the lack of everything needed for well-being (experience of a good quality of life). Poverty has many dimensions: physical, social, economic, political, psychological, and spiritual, including the vulnerabilities suffered by the poor, such as physical weakness, isolation, vulnerability, and powerlessness [3]. As a result, poor people in urban areas are more susceptible to illness. This situation is a double burden due to poor environmental quality and poverty [4].

About 7% of the urban population in Indonesia is poor, almost above the poverty line [5]. In congested conditions, the imperfect urban lives near open sewers and standing water are constantly exposed to harmful waste. Inadequate sanitation can lead to the transmission of worms and other intestinal parasites. The urban poor has limited financial resources and higher food costs, so they often experience a lack of nutritious food. Environmental pollution and food often prepared under unhygienic conditions cause disease [6, 7]. In illness, the urban poor must face shocks that impact their limited assets, livelihoods, and savings [5].

The main urban problems in developing countries show two interacting subsystems, namely: 1) Social subsystems such as unemployment, scarcity of access to clean water and sanitation, health services), and; 2) Environmental subsystem (water, air, and soil pollution, waste management, scarcity of clean water, settlements). The needs of the urban poor to survive in good health are often unmet because of the difficulty of accessing public services [3]. The majority of the poor population in urban areas live in temporary buildings. They made it of temporary materials such as bamboo, wood, corrugated zinc, and straw. Vulnerability in these settlements is further exacerbated by the lack of essential services and the disaster hazard stemming from natural disasters [8, 9].

The research results on poor people in Jakarta's slum areas show that they choose to move to Jakarta because they have lost their work relationships and hope Jakarta will provide a better life than their original area. Most work as laborers or porters or porters. Because their income was insufficient to rent a house, the situation forced them to move under the bridge. Environmental sanitation problems become expensive to obtain, and all places around the house can be trash cans [10]. Previous studies reported that urban residents living in slums said sick household members for two weeks [2]. Meanwhile, other studies state that the most vulnerable groups in informal urban settlements are toddlers, children, the elderly, and people with low immune systems [11, 12].

Apart from being susceptible to illness, the condition of urban poor is also vulnerable to becoming poorer. Previous studies have concluded that living in urban areas is associated with a higher likelihood of being poor [13]. Living in urban areas comes with a high price tag, making people more vulnerable to poverty. Prices of goods in urban areas are more expensive and susceptible to fluctuations. Therefore, they put vulnerable people at risk of falling into poverty if their wages are insufficient to cover the cost of living. Moreover, urban needs are more complex than those in rural areas [14]. So to survive, the poor in urban areas must continue working, even when sick.

People with low economic status often cannot meet their health needs; therefore, the Government of Indonesia launched the National Health Insurance (NHI) in 2014. The government has expanded the contribution subsidy for underprivileged families [15]. This National Health Insurance Policy aims to improve the quality of health services and be able to be reached all groups, especially the lower middle class. As the implementer of the policy, Health Social Security Administrator (SSA) has made maximum efforts to provide socialization and increase public awareness of the importance of health insurance. Still, guaranteeing national health insurance takes a process and time [16].

A previous study has reported that pro-poor interventions such as the national social health insurance scheme have been shown to have contributed to the quality of health services [17]. If successful, the NHI will become the most extensive single-payer system globally. Indonesia has made steady progress, but about a third of its population remains without protection and direct payments for health, even among NHI members. To help close this gap, particularly among the poor, the Indonesian government is implementing a series of Universal Health Coverage (UHC) policy reforms that include the integration of the government's remaining insurance schemes into the NHI [18]. UHC achievement is realized through participation in NHI by all Indonesian people, without exception. The local government registered the poor and underprivileged and paid as a participant in the Contribution Assistance Recipient (CAR) [19]. Based on the background narration, the study aims to analyze the effectiveness of government-run insurance in hospital utilization in urban poor in Indonesia.

## Materials and methods

### Data source

The study looked at secondary data from the Indonesian Basic Health Survey from 2018, and the Ministry of Health of the Republic of Indonesia conducted a national-scale cross-sectional survey. Furthermore, the study collected data from May to July 2018 through interviews with Household Instruments and Individual Instruments [20].

The 2018 Indonesian Basic Health Survey's population included all Indonesian households. The survey took the sample structure from the 2018 National Socio-Economic Survey, which was performed in March 2018. Furthermore, the survey visited 300,000 families from 30,000 census blocks in the 2018 Socio-Economic Survey (run by the Central Statistics Agency) [20].

The survey used the PPS (probability proportional to size) approach, employing systematic linear sampling in two stages: Stage 1: Implicit stratification based on welfare strata of all census blocks resulting from the 2010 Population Census. PPS chose the sample survey as the sampling frame for selecting census blocks from a master frame of 720,000 from the 2010 Population Census, of which 180,000 were chosen (25%). The survey used the PPS method to determine numerous census blocks in each urban/rural strata per regency/city to create a Census Block Sample List,. There are 30,000 Census Blocks in total that have been chosen. Stage 2: Using systematic sampling, select ten homes in each Census Block with the highest implicit stratification of education completed by the Head of Household to preserve the representation of the diversity value of household characteristics. All household members in the selected household will be questioned as part of the 2018 Indonesian Basic Health Survey [20].

The participants were all adults (aged 15 and up) living in Indonesia's impoverished urban societies. The study defined 75,970 people as a weighted sample based on the sampling procedures.

### Setting

The study setting was the urban poor in Indonesia at the national level. The study used the Indonesian Central Statistics Agency's provisions for urban categorization in the survey. The survey employed the wealth index formula to determine wealth status. The survey calculated the wealth index by taking a weighted average of a family's overall spending. Meanwhile, the poll used household expenditures such as health insurance, food, and accommodation to calculate the wealth index. Moreover, the survey divided the income index into five categories: quintile 1 (poorest), quintile 2 (poorer), quintile 3 (middle), quintile 4 (richer), and quintile 5 (richest) [21, 22]—the study categories quintile 1 and 2 as the poor.

### Outcome variable

The study's outcome variable was hospital utilization, which included adults' access to outpatient or inpatient hospitals. We had unutilized and utilized hospital beds in hospital utilization (outpatient and inpatient). Outpatient was limited to the preceding month, while inpatient was limited to the previous year. The survey asked respondents to recall outpatient and inpatient episodes appropriately depending on the time reference [20].

### Exposure variable

The study included health insurance ownership as an exposure variable. The study categorized health insurance ownership into four categories: uninsured, government-run insurance, private-run insurance, and a combination of both insurances (government-run and private-run insurance) [20].

### Control variables

The study employed five elements as control variables as part of such factors. The five criteria were age, gender, marital status, educational level, and occupation type.

The study calculated the respondent's age using the respondent's last birthday. However, we separated gender into two categories: male and female. In addition, the study divided marital status into three categories: never married, married/living with a partner, and divorced/widowed.

The acknowledgment of the respondent's most recent diploma is their education. The survey covers four levels of education: none, primary, secondary, and higher. Meanwhile, there are six occupations: no job, government employee/army/police, private sector, entrepreneur, farmer/fisherman/laborer, and others.

### Data analysis

The Chi-Square test was used in the early phases of the sample to create a bivariate comparison for the dichotomous variable. We also used the T-test for the continuous variable in the study (age). The study also used a collinearity test to check that the independent variables in the final regression model did not have a strong relationship. The analysis used a binary logistic regression at the study's last point. We employed the previous test to examine the multivariate link between all independent factors and hospital utilization. Throughout the statistical analysis portion of the investigation, we used the IBM SPSS 26 application.

### Ethics approval and consent to participate

The National Ethics Committee in the National Institute of Health Research and Development granted the 2018 Indonesian Basic Health Survey (LB.02.01/2/KE.024/2018). The survey deleted all respondents' identities from the dataset. Respondents have provided written approval for their involvement in the study. The author has obtained permission to use data for this study through the website: http://www.litbang.kemkes.go.id/layanan-permintaan-data-riset/.

## Results

The analysis found that Indonesia's national average hospital utilization among the urban poor in 2018 was 4.2%. Meanwhile, the distribution of health insurance ownership among urban-poor communities was uninsured at 34.9%, government-run insurance at 63.1%, private-run insurance at 1.6%, and simultaneous government-run and private-run insurance at 0.4%.

Table 1 shows descriptive statistics of the participants. Based on hospital utilization, unutilized people are mainly in all types of health insurance ownership. Meanwhile, those with government-run insurance have an older average age than those with other types of health insurance ownership. Moreover, based on gender, females lead in uninsured and government-run insurance; males lead in private-run and combined government-run and private-run insurance.

**Table 1:** Descriptive statistic of participants (*n* = 75,970)

Participants Characteristics	Health Insurance Ownership				*P*-value
	**Uninsured** **(*n* = 24,534)**	**Government-run** **(*n* = 50,233)**	**Private-run** **(*n* = 938)**	**Government-run and Private-run** **(*n* = 265)**	
**Hospital utilization**					< 0.001
Unutilized	98.6%	94.3%	93.9%	87.3%	
Utilized	1.4%	5.7%	6.1%	12.7%	
**Age (mean)**	(39.47)	(40.71)	(35.55)	(37.75)	< 0.001
**Gender**					
Male	49.2%	48.4%	51.5%	52.7%	
Female	50.8%	51.6%	48.5%	47.3%	
**Marital status**					< 0.001
Never in union	22.9%	23.6%	20.9%	23.0%	
Married/living with a partner	67.6%	67.1%	75.2%	67.2%	
Divorced/Widowed	9.5%	9.2%	3.9%	9.8%	
**Education level**					< 0.001
No education	6.0%	6.5%	1.0%	4.6%	
Primary	65.9%	66.6%	40.3%	48.6%	
Secondary	25.5%	24.4%	52.8%	43.9%	
Higher	2.6%	2.6%	5.8%	2.9%	
**Work type**					< 0.001
No job	42.6%	42.6%	32.9%	26.0%	
Civil servant/army/police	0.3%	0.7%	1.6%	2.1%	
Private sector	6.2%	7.7%	29.8%	32.4%	
Entrepreneur	16.9%	13.8%	11.7%	11.3%	
Farmer/fisherman/labor	28.1%	29.8%	18.9%	23.7%	
Others	5.8%	5.4%	5.0%	4.4%	

Regarding marital status, they are married or living with a partner dominated in all kinds of health insurance ownership. On the other hand, based on education level, primary education leads to uninsured, government-run insurance, and combined government-run and private-run insurance; meanwhile, secondary education leads to private-run insurance. Furthermore, no job leads to all types of health insurance ownership according to occupation type.

The study performed a collinearity test for the next stage. According to the outcome, each factor's variance inflation factor (VIF) value is less than 10.0, and the tolerance value for each variable is more significant than 0.10. The research suggests the test's decision-making basis since the regression model exhibited no multicollinearity.

Table 2 explains the binary logistic regression of hospital utilization among the urban poor in Indonesia. Table 2 shows, based on health insurance ownership, someone with government-run insurance is 4.261 times more likely than the uninsured to utilize the hospital (AOR 4.261; 95% CI 4.238–4.285). Someone with private-run insurance is 4.866 times more likely than the uninsured to use the hospital (AOR 4.866; 95% CI 4.802–4.931). Moreover, someone with government-run and private-run insurance is 11.974 times more likely than the uninsured to utilize the hospital (AOR 11.974; 95% CI 11.752–12.200).

**Table 2:** The result of binary logistic regression of hospital utilization among the urban poor in Indonesia in 2018 (*n* = 75,970)

Predictor	*P*-value	Hospital Utilization		
		**AOR**	**95% Confidence Interval**	
			**Lower Bound**	**Upper Bound**
Insurance: Uninsured	-	-	-	-
Insurance: Government-run	< 0.001	4.261	4.238	4.285
Insurance: Private-run	< 0.001	4.866	4.802	4.931
Insurance: Government-run & Private-run	< 0.001	11.974	11.752	12.200
Age	< 0.001	1.018	1.018	1.018
Gender: Male	-	-	-	-
Gender: Female	< 0.001	1.109	1.105	1.114
Marital: Never in union	-	-	-	-
Marital: Married/Living with partner	< 0.001	1.860	1.848	1.872
Marital: Divorced/Widowed	< 0.001	1.431	1.417	1.444
Education: No Education	-	-	-	-
Education: Primary	< 0.001	1.249	1.239	1.258
Education: Secondary	< 0.001	1.633	1.620	1.647
Education: Higher	< 0.001	1.475	1.455	1.496
Occupation: no job	-	-	-	-
Occupation: civil servant/army/police	< 0.001	0.485	0.474	0.497
Occupation: private sector	< 0.001	0.483	0.478	0.487
Occupation: entrepreneur	< 0.001	0.619	0.615	0.623
Occupation: farmer/fisherman/labor	< 0.001	0.471	0.469	0.474
Occupation: Others	< 0.001	0.784	0.778	0.790

*AOR* Adjusted Odds Ratio

In addition to health insurance ownership, the study found five control variables significant to hospital utilization among the urban poor in Indonesia: Age, gender, marital status, education level, and occupation type.

Based on gender, female is 1.109 times more likely to utilize the hospital than males (AOR 1.109; 95% CI 1.105–1.114). Regarding marital status, those married or living with a partner are 1.860 times more likely than those who never married to use the hospital (AOR 1.860; 95% CI 1.848–1.872). Moreover, those divorced or widowed are 1.431 times more likely than those who never married to utilize the hospital (AOR 1.431; 95% CI 1.417–1.444).

According to education level, all kinds of education have more possibilities to utilize the hospital than no education. On the other hand, based on occupation type, all occupations have a lower chance than no job to use the hospital.

## Discussion

The policy for implementing NHI in Indonesia has stated in Presidential Regulation of the Republic of Indonesia Number 82 of 2018 that health protection guarantees target the entire population without exception (universal coverage). The implementation of universal health protection coverage is given to every person (participant) who contributes, further divided into two, namely Contribution Assistance Recipient (CAR), which includes the poor and the needy non-CAR participants [23–25].

The results show someone with government-run insurance is more likely than the uninsured to utilize the hospital. Meanwhile, someone with private-run insurance is more likely to use the hospital than the uninsured. Moreover, someone with government-run and private-run insurance is more likely than the uninsured to utilize the hospital. In addition to guaranteed financing from participation in health insurance, hospitals' comprehensive services and facilities (referral services, inpatient care, emergency services) are driving factors for individuals choosing the type of health service facility. Private insurance usually plays a role by providing excess or additional coverage for services that are not included in government-run insurance [26, 27]. The study results align with previous studies, which show that the urban poor with insurance is more likely to take advantage of hospital services [28, 29]. This pattern is the same in the general population who are non-urban poor, both in Indonesia and other countries such as India and Ghana [4, 30, 31].

In general, poor people are less likely to have private insurance, although this does not rule them out, especially for those with chronic illnesses. Even though the Indonesian government has issued a CAR policy to cover the poor in NHI, there are several crucial issues that the government must address. For example regarding the quality of service that is considered lower, service that takes longer, or procedures that are difficult to understand. Resolving these issues can potentially increase NHI utilization [27, 32, 33].

The study found age is related to hospital utilization among the urban poor in Indonesia. People in urban, poor, and old age groups have more excellent opportunities to use hospitals as healthcare facilities. These results can explain that the catastrophic disease, which increases with age, often causes a person to require referral care. Furthermore, old age with low-income characteristics and unhealthy lifestyles will potentially have more diseases and body disorders. This condition can increase the risk of needing care compared to the elderly in social groups with better economic conditions [28, 34].

The result indicates female is more likely to utilize the hospital than males. This study's results align with the previous research [35, 36]. One thing that might be explained is related to the perception that is followed by health behavior from the side of the individual woman. Women who live less healthy lives than men are more likely to take advantage of the hospital, although biologically, women generally live longer than men [1, 37].

The study shows all marital statuses have more likely than those never in a union to use the hospital. This result supports the statement that marital status is an essential predictor of hospital utilization [38]. A study on colon cancer patients in Norway showed a similar effect that revealed more married or ever-married patients used health care in hospitals than those who never married [39]. In addition, other evidence from health insurance beneficiaries in the US and Puerto Rico. The studies reported that married people increase the likelihood of using health services efficiently compared to currently not-married people because they tend to choose outpatient care or skilled nursing facility [38]. The concept of married couples' protective role may explain this result. Spouses can provide physical and emotional support, including taking or accompanying their partner to a health facility for consultation or treatment [40, 41].

Moreover, marriage facilitates health budget-sharing engagement, allowing them to seek health care at health facilities [38, 42]. Those who are not married tend to be more prone to health problems [41]. Policymakers must understand health facilities utilization according to marriage status because the marriage number in Indonesia has declined in the last decade, from 2.3 million in 2011 to 1.7 million in 2021 [43]. It may affect health-seeking behavior, health status, and health budget.

The study informs all kinds of education have more possibilities to utilize the hospital than no education. Education is strongly associated with better income and wealth [44], and it can lead to better access to information, health insurance, and health. The Wisconsin cohort study also confirms that adults with higher educational attainment have more access to health care, particularly for preventive care, such as physical and dental checkups, flu vaccinations, and cholesterol tests [45, 46]. This behavior can accelerate positive health outcomes and increase healthcare costs as a short-term impact [46]. Therefore, policies related to health investment should align with intervention strategies to improve education, prioritizing the dropped out-of-school group for better health literacy.

Moreover, based on occupation type, all occupations have a lower chance than no job to use the hospital. A study in Sweden may explain that employed adults have a higher health-related quality of life scores than those unemployed [47]. This condition may prevent them from using hospitals or other health facilities frequently. Contrarily, being unemployed harms health, such as mental health [48]. A review study concluded that the unemployed was associated with increased healthcare utilization because universal health coverage was available and accessible for this group [49]. The Indonesian government has launched a national health insurance program, Healthy Indonesia Card (HIC), and poor people receive a beneficiary contribution scheme [50]. In 2016, this program protected 14.5 million poor people from severe poverty due to health care costs [51]. The program should run parallel with other social protection programs to achieve a sustainable impact targeting the poor or unemployed, such as business assistance for the working-age or small business loans.

### Strength and limitation

The study examines a large amount of data to represent information nationally. On the other hand, the study examines secondary data; therefore, the variables analyzed are limited to acceptable ones. Other variables linked to hospital utilization reported in previous studies, such as trip time, hospital travel costs, and disease type, cannot be investigated [4, 52, 53].

## Conclusion

Based on the results, the study concluded that government-run insurance is more effective than the uninsured in improving hospital utilization in urban poor in Indonesia. On the other hand, private-run is more effective than government-run and uninsured in improving hospital utilization in urban-poor communities in Indonesia. Furthermore, the most effective is to combine the kind of health insurance ownership (government-run and private-run).

## Data Availability

The author cannot publicly share the data because a third party and the Ministry of Health of the Republic of Indonesia, who owns the data, do not have permission to share it. The 2018 Indonesian Basic Health Survey data set is available on the web http://www.litbang.kemkes.go.id/layanan-permintaan-data-riset/ for researchers who meet the criteria for access to confidential data.

## Abbreviations

NHI: National Health Insurance
UHC: Universal Health Coverage
SDGs: Sustainable Development Goals
PPS: Probability proportional to size
VIF: Variance inflation factor
AOR: Adjusted odds ratio
